# The Value of Platelet-to-Lymphocyte Ratio (PLR) in Identifying Intracranial Injury in Patients with Mild Head Trauma: A Prospective Study

**DOI:** 10.3390/jcm15114052

**Published:** 2026-05-24

**Authors:** Sedat Özbay, Ökkeş Zortuk, Yavuz Fatih Yavuz, Cemil Kavalcı, Taha Yaşar Kiraz, Orhan Özsoy, Tansu Gençer

**Affiliations:** 1 Department of Emergency Medicine, Sivas Numune Hospital, Sivas 58060, Turkey; ilsedat58@hotmail.com (S.Ö.); rhnzsy@gmail.com (O.Ö.); tansu_dalar@hotmail.com (T.G.); 2 Department of Emergency Medicine, Burdur Mehmet Akif Ersoy University, Burdur 15030, Turkey; 3 Department of Emergency Medicine, SBU Antalya Research and Training Hospital, Antalya 07100, Turkey; yavuzfatihyavuz@gmail.com (Y.F.Y.); cemkavalci@yahoo.com (C.K.); dr.tahakiraz@gmail.com (T.Y.K.)

**Keywords:** emergency medicine, mild head trauma, PLR

## Abstract

**Background**: Head trauma is a major public health concern. Computed tomography (CT) is frequently used to evaluate these patients but may expose them to unnecessary radiation exposure. Various biomarkers have been investigated to predict prognosis and reduce the need for unnecessary imaging. Red cell distribution width (RDW), neutrophil/lymphocyte ratio (NLR), and platelet/lymphocyte ratio (PLR) have been proposed as inflammatory markers; however, their diagnostic value in head trauma remains controversial. This study aimed to determine the value of complete blood count parameters in identifying intracranial injury in patients with mild head trauma. **Methods**: This prospective, single-center study enrolled 100 adults with mild head trauma. Demographic data, vital signs, neurological assessments, complete blood counts, CT results, and clinical outcomes were also recorded. Patients were categorized as intracranial injury positive (Group 1) or intracranial injury negative (Group 2). We statistically compared the laboratory and demographic data of the groups. Statistical significance was set at *p* < 0.05. **Results**: The study included 100 patients with mild head trauma who presented to the emergency department, of whom 11 were in Group 1. The median PLR and lymphocyte levels differed significantly between the groups (*p* < 0.05). **Conclusions**: The PLR may serve as a preliminary supportive marker to aid clinical assessment; however, its modest discriminatory performance suggests that it should not be used as a standalone diagnostic tool.

## 1. Introduction

Head trauma is a major global health problem and remains one of the leading causes of emergency department (ED) visits. Mild head trauma represents approximately 80% of all head injuries and is defined by a Glasgow Coma Scale (GCS) score of 15, transient loss of consciousness of <20 min, and absence of focal neurological deficits [1,2,3]. Although most of these patients do not develop clinically significant intracranial pathology, the high frequency of presentations makes efficient and accurate risk stratification essential in contemporary ED practice.

Computed tomography (CT) is the gold standard for evaluating intracranial injuries in patients with mild head trauma. However, routine CT use is challenged by concerns regarding radiation exposure, increased cost, ED crowding, and low diagnostic yield in this patient group [3,4]. These limitations underscore the need for complementary, accessible, and reproducible biomarkers to support clinical decision-making and reduce unnecessary imaging.

In this context, hematological indices derived from complete blood counts, such as the neutrophil-to-lymphocyte ratio (NLR), platelet-to-lymphocyte ratio (PLR), red cell distribution width (RDW), mean platelet volume (MPV), and platelet count, have been investigated as potential indicators of inflammatory activation. Their biological relevance stems from the established role of post-traumatic inflammation in secondary brain injury, which contributes to neuronal damage following the initial mechanical insult [1,5,6]. Given their widespread availability, low cost, and rapid turnaround time, these markers hold promise as adjunctive tools for the early assessment of mild head injuries.

Despite the growing interest, current evidence regarding the prognostic value of NLR, PLR, and RDW in predicting intracranial injury in patients with mild head trauma remains inconsistent. Some studies have reported significant associations, whereas others have demonstrated no meaningful correlation or focused primarily on moderate-to-severe traumatic brain injury. Heterogeneity in study design, timing of blood sampling, and variability in cutoff values further contribute to these discrepancies. Consequently, the predictive utility of routinely available hematological biomarkers in this patient population remains uncertain [5,7,8].

In this study, we specifically focused on patients with a Glasgow Coma Scale (GCS) score of 15. While mild traumatic brain injury is traditionally defined as GCS 13–15, patients with GCS 13–14 are almost universally scanned according to clinical protocols. The clinical dilemma and risk of unnecessary CT imaging are most prominent in the GCS 15 group, where the decision to image often relies on more subtle clinical findings.

Addressing this gap is essential for determining whether these markers can enhance diagnostic algorithms and optimize CT utilization. Therefore, this study aimed to determine the value of complete blood count parameters in identifying intracranial injury in patients with mild head trauma.

## 2. Materials and Methods

### 2.1. Study Design and Ethical Considerations

This prospective study was conducted at a tertiary training and research hospital following approval from the local ethics committee (Decision No 2/13). All procedures adhered to the principles of the Declaration of Helsinki. Written informed consent was obtained from all participants prior to enrollment.

Patients were categorized into two groups based on their CT head findings. Group 1 (intracranial injury positive) included patients with intracranial hemorrhage, cerebral contusion, traumatic brain edema, traumatic linear fractures, and isolated linear skull fractures. Group 2 (intracranial injury-negative patients) included patients with normal head CT findings and no intracranial injury.

### 2.2. Participants

Consecutive adult patients (≥18 years) presenting to the emergency department with mild head trauma were screened. Mild head trauma was defined as a Glasgow Coma Scale (GCS) score of 15, loss of consciousness lasting <20 min, and absence of focal neurological deficits on initial examination.

The inclusion criteria for this study required that participants be 18 years of age or older, present with a mild head injury meeting the defined criteria, and have undergone both blood sampling and CT imaging at the time of admission. Conversely, patients under 18 years of age, those with a GCS score of 13 or lower, those with a GCS score of 14 who exhibited neurological deficits, those with moderate/severe head trauma, those currently receiving anticoagulant or antiplatelet therapy, those with a history of known intracranial mass or prior intracranial surgery, and those with a diagnosed hematological disorder were excluded from the study.

### 2.3. Data Collection

Demographic characteristics, vital signs (systolic/diastolic blood pressure and heart rate), GCS scores, and clinical outcomes (hospitalization vs. discharge) were recorded.

### 2.4. Blood Biomarker Measurement

Venous blood samples were collected upon admission, preferably within 6 h of the injury. Samples were drawn into standard EDTA tubes and processed immediately. Complete blood count (CBC) parameters, including neutrophil, lymphocyte, and platelet counts, NLR, PLR, and RDW, were analyzed using an automated hematology analyzer (Sysmex XN-1000; Sysmex Corporation, Kobe, Japan) according to the manufacturer’s protocols. Internal and external quality control procedures were routinely performed.

Variables with *p*-values < 0.10 in the univariate analysis (including lymphocyte count, NLR, PLR, RDW, and sex) were entered into the multivariable logistic regression model to identify independent predictors of intracranial injury.

### 2.5. CT Imaging and Interpretation

Non-contrast head CT scans were performed in all patients during the initial evaluation period. The images were reviewed by a board-certified radiologist who was blinded to the results. Any discrepancies in interpretation were resolved by consensus with a second radiologist or, when necessary, by a senior radiologist.

The radiologists who evaluated the CT scans were blinded to the patients’ laboratory results and clinical data. Similarly, the laboratory staff were blinded to radiological findings.

### 2.6. Clinical Outcomes

Clinical outcomes included the need for hospitalization, defined as admission for observation, management of intracranial injury, or neurosurgical evaluation, and were recorded for each patient. This pragmatic clinical endpoint was recorded to complement the CT findings, acknowledging that institutional practices and clinicians’ judgment may influence admission decisions.

### 2.7. Sample Size

A minimum sample size of 100 patients was estimated based on the intracranial injury prevalence of 8.4% reported by Geritsen et al. [9], with a 95% confidence interval (CI) and a 5% margin of error. For a moderate effect level of 80% power and a *p*-value of <0.05, the number of patients required was calculated to be 100. The final study included 100 patients, consistent with the planned sample size.

### 2.8. Statistical Analysis

Data were analyzed using SPSS software (version 27; IBM Corp., Armonk, NY, USA). Categorical variables were summarized using frequency and percentage. The normality of the continuous variables was assessed using the Kolmogorov–Smirnov test. Normally distributed variables are presented as mean ± standard deviation (SD), whereas non-normally distributed variables are reported as medians and interquartile ranges (IQR).

Between-group comparisons were performed using the chi-square test for categorical variables and either the independent samples *t*-test or the Mann–Whitney U test for continuous variables, depending on their distribution. To assess the diagnostic performance of the PLR in determining intracranial injury, a receiver operating characteristic (ROC) curve was generated, and the area under the curve (AUC) was calculated. Multivariate logistic regression analysis was performed to examine the factors that may have an impact on intracranial injury. Variables with a *p*-value of <0.10 in the single model were transferred to multiple models. Statistical significance was set at *p* < 0.05. Statistical significance was set at *p* < 0.05.

## 3. Results

A total of 100 patients presenting with mild head trauma were included in the study and were categorized according to head CT findings as intracranial injury positive (group 1, n = 11) and intracranial injury negative (group 2, n = 89). A significant sex imbalance was observed between the groups, with males accounting for 90.9% of Group 1 compared with 56.2% in Group 2 (*p* = 0.027). The demographic and baseline clinical characteristics of the patients are presented in Table 1.

The median age was 33 years (IQR: 21) in Group 1 and 40 years (IQR: 22) in Group 2, with no statistically significant difference between the groups (*p* > 0.05). Males constituted 90.9% and 56.2% of Groups 1 and 2, respectively. Traffic accidents were the most common trauma mechanism among CT-positive patients, and the distribution differed significantly between groups (*p* < 0.001). In Group 1 (n = 11), the intracranial pathologies included hemorrhage (n = 9), cerebral contusion (n = 2), and isolated linear fractures (n = 10). Some patients sustained multiple injuries simultaneously, which accounted for the overlap in these counts. Vital signs, Glasgow Coma Scale scores, and hospitalization rates were comparable across groups (all *p* > 0.05). Regarding laboratory parameters, the PLR was significantly lower in Group 1 than in Group 2, whereas the lymphocyte count was significantly higher in Group 1 (*p* < 0.05). No significant differences were observed in neutrophil count, NLR, RDW, or platelet count (all *p* > 0.05).

**Figure 1:**
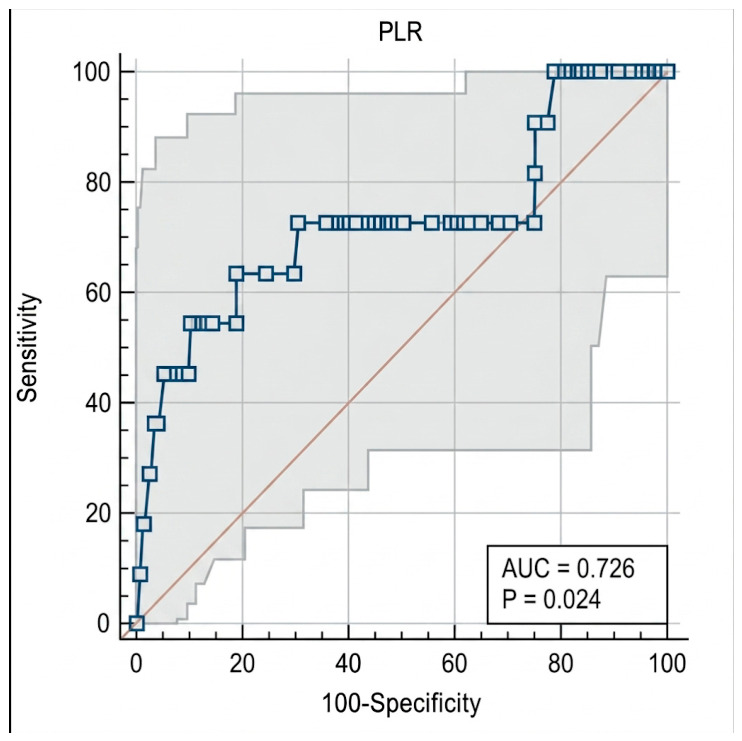
ROC analysis for definition of intracranial injury.

ROC analysis demonstrated a moderate diagnostic performance of PLR for predicting intracranial injury, with an AUC of 0.726 (95% CI, *p* = 0.024). At a cutoff value of <80.24, PLR yielded a sensitivity of 63.64%, specificity of 80.90%, PPV of 29.2%, and NPV of 94.7% (Figure 1).

Multivariate logistic regression analysis was performed to examine the factors that may have an impact on intracranial injury (Table 2). No single variable was found to be effective in determining intracranial injuries.

## 4. Discussion

In this prospective, single-center study, we investigated the role of hematological biomarkers in detecting intracranial injury in patients with mild head trauma. A statistically significant difference was observed in the lymphocyte levels and PLR between the groups. However, no statistically significant differences were observed in the other variables between the groups.

In our study, we observed that the PLR can be used as a supplementary tool for predicting intracranial injury. For values below 80.24, the specificity was 80.90%, while the sensitivity was 63.64%, the PPV was 29.2%, and the NPV was 94.7% (AUC = 0.726, *p* = 0.024).

Moreover, elevated PLR has been associated with the development of progressive hemorrhagic injury (PHI) in patients with TBI, which further impacts neurological prognosis [10]. A low PLR is an independent risk factor for mortality in traumatic brain injury, with significant survival differences between patients with low and high PLR levels [11]. In pediatric trauma, a higher PLR has been associated with better survival outcomes [12]. Acar et al. reported that PLR did not provide detailed information regarding its impact on TBI outcomes [13]. In our study, we observed that PLR may serve as a preliminary supportive marker for determining intracranial pressure in patients with mild head trauma. For values below 80.24, the specificity was 80.90%, while the sensitivity was 63.64%, PPV was 29.2%, and NPV was 94.7% (AUC = 0.726, *p* = 0.024). ROC analysis revealed a low PPV and high NPV for PLR, suggesting that PLR alone is insufficient for reliably detecting intracranial injury. The PLR is a valuable and readily available marker that can help identify high-risk trauma patients early, facilitating timely and targeted interventions. To establish the diagnostic value of PLR in patients with mild head trauma, further multicenter prospective studies involving more CT-positive patients are required.

In the follow-up of intracranial events, blood parameters can serve as prognostic indicators of mortality and morbidity [14,15,16]. Saburi et al. reported increased neutrophil counts and elevated NLR values following brain injury [14]. Furthermore, several studies have highlighted the neutrophil count as a significant parameter [15,16]. In our study, neutrophil count and NLR were not significant predictors of minor head trauma, possibly because the degree of brain injury in this population was relatively mild.

Previous research on RDW in patients with head injury has produced conflicting results [5,6,17,18]. Some researchers have reported that RDW is associated with mortality in patients with head trauma [17,18]. Lin et al. found that elevated RDW is an independent risk factor for hospital and 6-month mortality in patients with moderate-severe TBI, but no significant correlation was observed in patients with mild TBI [19]. Consistent with Lin et al., our findings suggest that RDW is not a reliable marker for identifying intracranial injury in patients with mild head trauma.

In our study, contrary to some studies focusing on moderate-to-severe TBI, the PLR was significantly lower in the intracranial injury group. This finding was primarily driven by the significantly higher lymphocyte counts observed in Group 1 patients at the time of admission (*p* = 0.009). While trauma typically induces lymphopenia as part of a delayed stress response, the very early phases of acute injury can sometimes trigger a transient increase in circulating lymphocytes due to the immediate release of catecholamines and rapid recruitment of marginalized cells. This early ‘lymphocytosis of stress’ may explain why PLR appears lower in our injury group, especially given that blood samples were collected within the first 6 h of trauma.

The predominance of male patients in the intracranial injury group (Group 1) is consistent with the trauma literature, which often reports a higher incidence of traumatic brain injury and road traffic accidents among males. However, this sex imbalance represents a potential confounding factor, as physiological inflammatory responses and baseline hematological indices can vary between sexes.

Although the PLR demonstrated a high negative predictive value (94.7%) in the univariate analysis, its lack of independent predictive value in the multivariate analysis—likely due to the limited number of positive cases (n = 11)—precludes its use as a tool to definitively rule out CT imaging. Instead, it should only be interpreted as a preliminary, descriptive marker that requires validation in larger cohorts.

In the emergency department, several approaches are employed to predict life-threatening and morbid conditions. Easily obtainable complete blood count parameters may aid in the evaluation when a CT is not available. Our results suggest that while PLR values differ between groups on a univariable level, these changes are insufficient for standalone diagnostic screening or definitive clinical decision-making following mild head trauma.

## 5. Conclusions

In conclusion, the PLR does not possess independent diagnostic value for identifying intracranial injury in mild head trauma. It should not be used to guide or omit CT scan use in the emergency department, but may only serve as a weak, supplementary marker whose true utility must be evaluated in larger prospective studies with higher CT-positive patient volumes.

## 6. Limitations

This study had several limitations. First, it was conducted at a single center, which may limit the generalizability of our findings. Second, the relatively small sample size, particularly the limited number of Group 1 patients (n = 11), reduced the statistical power for multivariable analyses and increased the risk of type-II error.

Another limitation is the significant sex disparity between the study groups. Given that the majority of patients with intracranial injury were male, our findings regarding the PLR may not be fully generalizable across genders, and sex could serve as a confounding variable in the inflammatory response to trauma.

## Figures and Tables

**Table 1:** Comparison of demographic and Laboratory findings regarding groups.

Variable	Group 1 (n = 11)	Group 2 (n = 89)	*p*-Value
Gender, male/female (n, %)	10/1	50/39	0.027
Age (median IQR)	33 (21)	40 (22)	0.119
Mortality (n, %)	0 (0%)	3 (3.37%)	0.536
CT finding			
Normal (n, %)	0	79 (88.76%)	<0.001
Hemorrhage (n, %)	9 (81.81)	0	
Contusion (n, %)	2 (22.22%)	0	
Isolated Linear Fracture (n, %)	10 (90.90%)	0	
WBC (10^3^/mikroL)	12.90 (9.80)	10.90 (7.90)	0.315
Plt (10^3^/mikroL)	260 (124)	224 (75)	0.206
Neutrophil (10^3^/mikroL)	8.70 (6.50)	7.06 (5.33)	0.346
Lymphocyte (10^3^/mikroL)	4.05 (4.24)	2.20 (1.78)	0.009
NLR	2.46 (1.92)	3.47 (3.09)	0.073
PLR	69.56 (102.65)	112.50 (64.09)	0.015
RDW (mikrometer)	44.80 (8.90)	41.30 (6.10)	0.069

CT: Computed tomography, WBC: White Blood Count, Plt: Platelet, Neu: Neutrophile, Lym: lymphocyte, NLR: Neutrophile/lymphocyte ratio, IQR: interquartile range, RDW: Red Cell Distribution Width Note: Some patients presented with multiple intracranial pathologies simultaneously (e.g., both hemorrhage and contusion), so the total number of findings may exceed the number of patients in Group 1.

**Table 2:** Logistic regression analysis for intracranial injury.

Variable	Odds Ratio	*p*-Value	95% CI [Profile Likelihood]
Age	0.958	0.158	0.906 to 1.1017
WBC	0.919	0.444	0.741 to 1.140
PLT	1.000	0.742	1.000 to 1.000
RDW	1.017	0.837	0.866 to 1.194
Neu	1.257	0.090	0.965 to 1.638
Lym	1.055	0.578	0.874 to 1.273
NLR	0.629	0.186	0.317 to 1.250
PLR	1.000	0.987	0.995 to 1.005

WBC: White Blood Count, PLT: Platelet, RDW: Red Cell Distribution Width, Neu: Neutrophile, Lym: lymphocyte, NLR: Neutrophile/lymphocyte ratio, PLR: Platelet/lymphocyte ratio.

## Data Availability

The datasets used and/or analyzed during the current study are available for sharing by the corresponding author, upon request.

## Abbreviations

The following abbreviations are used in this manuscript.

Traumatic brain injury	[TBI]
Computerized Tomography	[CT]
Neutrophil-to-lymphocyte ratio	[NLR]
Platelet-to- lymphocyte ratio	[PLR]
Mean platelet volume	[MPV]
Red cell distribution width	[RDW]
Mild head trauma	[MHT]
